# Ultrasonographic Anterior Talofibular Ligament Thickness Does Not Predict Functional Outcomes After Acute Lateral Ankle Sprain

**DOI:** 10.1002/jfa2.70187

**Published:** 2026-07-20

**Authors:** Seung Myung Wi, Dong Hee Kim, Sang Heun Kim, Sung Jin Shin

**Affiliations:** ^1^ Department of Orthopedic Surgery Samsung Changwon Hospital Sungkyunkwan University School of Medicine Changwon Korea

**Keywords:** anterior talofibular ligament, lateral ankle sprain, ligament thickness, ultrasonography

## Abstract

**Background:**

Ultrasonography is commonly used to evaluate ligament injuries following acute lateral ankle sprain. However, the clinical significance of structural changes in ligament thickness during the healing process remains unclear. This study aimed to evaluate whether changes in anterior talofibular ligament (ATFL) thickness are associated with functional clinical outcomes.

**Methods:**

This study was a retrospective analysis of prospectively collected cohort data involving 32 patients with acute lateral ankle sprain enrolled between January 2024 and March 2025. All patients underwent conservative treatment and were followed for up to 12 weeks after injury. Patients were excluded if they had a history of prior ankle sprain, fracture, deformity, previous surgery involving the foot or ankle, or avulsion‐type ATFL injury. Standardized ultrasound examinations were performed at baseline and at 3, 6, and 12 weeks after injury. ATFL thickness was measured bilaterally at the ligament midpoint, and an injured‐to‐healthy thickness ratio was calculated. Functional outcomes were assessed at 12 weeks using the visual analog scale (VAS), American Orthopedic Foot and Ankle Society (AOFAS) score, and Cumberland Ankle Instability Tool (CAIT). Temporal changes were analyzed using repeated‐measures ANOVA, and correlations were assessed using Pearson correlation analysis.

**Results:**

The ATFL thickness ratio decreased significantly over time (*p* < 0.05), suggesting progressive structural remodeling during healing. Clinical outcomes improved significantly (all *p* < 0.001). However, no significant associations were detected between the ATFL thickness ratio and functional outcome measures at any time point (all *p* > 0.05).

**Conclusions:**

Although serial ultrasonography demonstrated progressive morphological remodeling of the ATFL following acute lateral ankle sprain, the present study did not detect a significant association between the ATFL thickness ratio and short‐term functional outcomes. These findings suggest that ultrasonographic thickness alone may not fully reflect clinical recovery.

## 1 Introduction

Lateral ankle sprain is one of the most common musculoskeletal injuries encountered in clinical practice and frequently affects physically active individuals [1]. Although most patients recover with conservative management, a substantial proportion experience persistent symptoms such as pain, instability, or recurrent sprains. Identifying factors that predict functional recovery after acute ankle sprain remains an important clinical challenge [2, 3].

The anterior talofibular ligament (ATFL) is the most commonly injured ligament in lateral ankle sprains and serves as a primary stabilizer against the internal rotation of talus in the mortis and inversion during plantar flexion [4, 5, 6, 7]. Imaging modalities such as magnetic resonance imaging (MRI) and ultrasonography are widely used to evaluate ligament injury [8, 9, 10, 11]. In particular, ultrasonography offers several advantages, including real‐time assessment, accessibility, and the ability to perform dynamic evaluation [10, 11, 12].

Previous studies have demonstrated that ligament thickening is a common finding following ankle sprain and may persist after healing [13, 14, 15]. However, the clinical significance of such morphologic changes remains unclear [1, 11]. Cross‐sectional studies have reported increased ATFL thickness in previously injured ankles, but similar degrees of thickening have been observed in both asymptomatic individuals and patients with chronic ankle instability [4]. These findings suggest that structural changes in ligament morphology may not directly correlate with functional outcomes.

Despite increasing use of ultrasonography in the evaluation of ankle sprains, there is limited clinical evidence regarding the relationship between serial sonographic changes in the ligament structure and clinical recovery. In particular, it remains uncertain whether changes in ATFL thickness during the healing process can serve as a prognostic indicator of functional outcomes.

Therefore, the purpose of this study was to evaluate the relationship between structural healing of the ATFL, assessed using serial ultrasonographic measurements, and functional clinical outcomes following acute lateral ankle sprain. Specifically, we aimed to determine whether the injured‐to‐healthy ATFL thickness ratio is associated with functional recovery and characterize the temporal relationship between structural changes and symptom improvement.

## 2 Methods

### 2.1 Study Design and Participants

This study was a retrospective analysis of prospectively collected cohort data involving 32 patients with acute lateral ankle sprain enrolled between January 2024 and March 2025. All patients underwent conservative treatment and were followed for up to 12 weeks after injury. Patients were excluded if they had a history of prior ankle sprain, fracture, deformity, or previous surgery involving the foot and ankle or avulsion‐type ATFL injury.

Patients were classified according to the severity of the ankle sprain. Injury severity was classified according to clinical and ultrasonographic findings based on criteria described in previous studies [16]. Grade 2 injuries were defined as partial ATFL tears with ligament continuity preserved, whereas Grade 3 injuries were defined as complete ligament disruption with instability findings. As no Grade 1 injuries were included in the final cohort, comparative analyses were performed between Grade 2 and Grade 3 sprains.

### 2.2 Ultrasound Assessment

**FIGURE 1:**
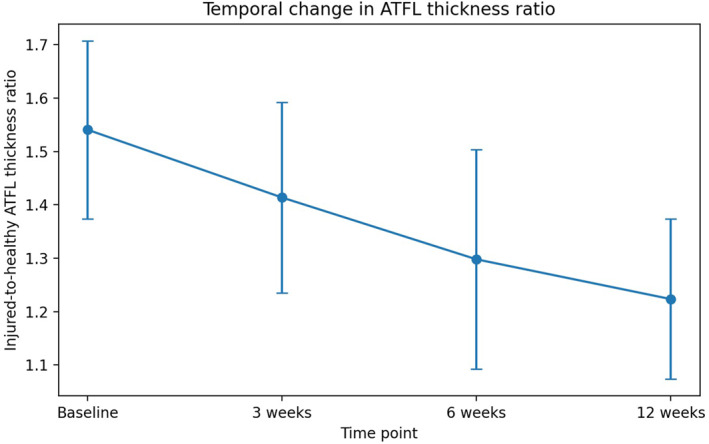
Representative serial ultrasonographic images of the anterior talofibular ligament (ATFL) obtained during follow‐up after acute lateral ankle sprain. Longitudinal images were acquired at baseline, 3 weeks, 6 weeks, and 12 weeks after injury (from left to right). Ligament thickness was measured at the midpoint of the ATFL (yellow markers). Progressive reduction in ligament thickness was observed during the healing process, suggesting structural remodeling over time.

Standardized ultrasound examinations were performed at the time of injury (baseline) and at 3, 6, and 12 weeks after injury. All examinations were conducted using a high‐frequency linear transducer (V8, Samsung Medison Co. Ltd., Seoul, Korea) with the ankle positioned in slight plantarflexion. All ultrasonographic measurements were performed by a single experienced examiner (S.J. Shin) to minimize interobserver variability. ATFL thickness was measured twice at each time point, and the mean value was used for analysis. Intraobserver reliability was assessed using the intraclass correlation coefficient (ICC) (Figure 1).

The anterior talofibular ligament (ATFL) was evaluated in the longitudinal plane. Ligament thickness was measured at the midpoint of the ATFL on both the injured and contralateral healthy ankles. To account for inter‐individual variability, an injured‐to‐healthy ATFL thickness ratio was calculated at each time point.

### 2.3 Clinical Outcome Assessment

Functional outcomes were evaluated at 12 weeks using the American Orthopedic Foot and Ankle Society (AOFAS) ankle–hindfoot score. Pain severity was assessed using a visual analog scale (VAS), and perceived ankle instability was evaluated using the Cumberland Ankle Instability Tool (CAIT) [17].

### 2.4 Conservative Treatment Protocol

All patients were managed using a standardized conservative treatment protocol, although minor adjustments were made according to swelling and pain tolerance. At the initial visit, a short leg splint was applied, and patients were instructed in the principles of protection, rest, ice, compression, and elevation (PRICE). At 1 week after injury, the splint was replaced with a short leg cast if swelling had sufficiently subsided, allowing for protected weight‐bearing as tolerated. Immobilization was maintained during the early phase to provide adequate pain control, reduce swelling, and protect the injured ligament during initial healing before progression to functional rehabilitation. At 3 weeks after injury, the cast was removed and an ankle brace was applied. Patients were then permitted to begin range‐of‐motion exercises and peroneal muscle strengthening. At 6 weeks after injury, the ankle brace was discontinued, and patients were encouraged to perform progressive muscle strengthening and return gradually to normal activities. Exercise progression was guided by patient tolerance. At 12 weeks after injury, patients were allowed to gradually return to physical activity.

### 2.5 Statistical Analysis

Temporal changes in the ATFL thickness ratio across time points (baseline, 3, 6, and 12 weeks) were analyzed using repeated measures analysis of variance (ANOVA). Changes in clinical outcome measures (VAS, AOFAS, and CAIT) between baseline and 12 weeks were analyzed using paired t‐tests. The relationships between ATFL thickness ratios and clinical outcomes at 12 weeks were evaluated using Pearson correlation analysis. Comparisons between Grade 2 and Grade 3 sprains were performed using the Mann–Whitney *U* test. A two‐tailed *p*‐value of < 0.05 was considered statistically significant.

## 3 Results

### 3.1 Patient Characteristics

A total of 32 patients were included in the final analysis, consisting of 17 patients with Grade 2 sprains and 15 patients with Grade 3 sprains. The mean age was 32.0 ± 11.8 years (range, 16–63 years), and the mean BMI was 23.2 ± 0.8 kg/m^2^ (Table 1).

**TABLE 1:** Baseline characteristics.

	Total (*n* = 32)
Age (years)	32
BMI (kg/m^2^)	23.2
Ankle sprain Grade 2	17
Ankle sprain Grade 3	15
VAS (12 weeks)	1.09 ± 1.38
AOFAS (12 weeks)	91.12 ± 8.20
CAIT (12 weeks)	27.25 ± 2.11

### 3.2 Temporal Changes in ATFL Thickness Ratio

**FIGURE 2:**
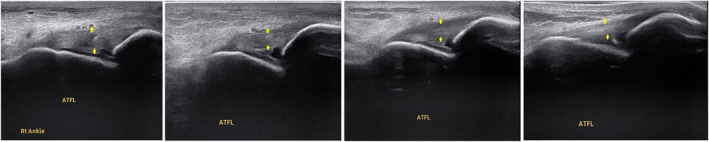
Temporal changes in the injured‐to‐healthy anterior talofibular ligament (ATFL) thickness ratio during the healing process following acute lateral ankle sprain. The ATFL thickness ratio progressively decreased from baseline to 12 weeks after injury, indicating structural remodeling over time. (Data are presented as mean ± standard deviation. Repeated‐measures ANOVA demonstrated a significant temporal change across follow‐up periods [*p* < 0.05]).

The injured‐to‐healthy ATFL thickness ratio progressively decreased over time, from 1.53 ± 0.17 at baseline to 1.42 ± 0.17 at 3 weeks, 1.30 ± 0.21 at 6 weeks, and 1.21 ± 0.14 at 12 weeks (Figure 2).

Pooled intraobserver ICC for all paired thickness measurements was 0.928, indicating excellent measurement reliability. Repeated‐measures ANOVA demonstrated a significant change in the ATFL thickness ratio across time points (*p* < 0.05), indicating progressive structural remodeling during the healing process.

### 3.3 Changes in Clinical Outcomes

Clinical outcomes improved significantly over the follow‐up period.

The mean VAS score decreased from 5.62 ± 0.71 at baseline to 1.09 ± 1.38 at 12 weeks. The mean AOFAS score increased from 59.44 ± 6.09 to 91.12 ± 8.20 and the mean CAIT score increased from 20.75 ± 1.46 to 27.25 ± 2.11. These changes were all statistically significant (paired *t*‐test, *p* < 0.001).

### 3.4 Correlation Between ATFL Thickness Ratio and Clinical Outcomes

Pearson correlation analysis revealed no statistically significant associations between ATFL thickness ratios at any time point and clinical outcome measures at 12 weeks, including VAS, AOFAS, and CAIT scores (all *p* > 0.05) (Table 2).

**TABLE 2:** Correlation between ATFL thickness ratio at each time point (baseline, 3 weeks, 6 weeks, and 12 weeks) and clinical outcomes at 12 weeks (VAS, AOFAS, and CAIT).

	VAS (12 weeks)	AOFAS (12 weeks)	CAIT (12 weeks)
Baseline (at the time of injury)	*p* = 0.310	*p* = 0.378	*p* = 0.505
3 weeks	*p* = 0.113	*p* = 0.171	*p* = 0.743
6 weeks	*p* = 0.104	*p* = 0.669	*p* = 0.450
12 weeks	*p* = 0.189	*p* = 0.904	*p* = 0.559

*Note:* No significant correlations were observed at any time point.

### 3.5 Comparison Between Grade 2 and Grade 3 Sprains

No significant differences in ATFL thickness ratios were observed between Grade 2 and Grade 3 sprains at any time point (all Mann–Whitney *U* test, *p* > 0.05). Furthermore, there were no significant differences in clinical outcomes at 12 weeks, including VAS, AOFAS, and CAIT scores, between the two groups (all *p* > 0.05) (Table 3).

**TABLE 3:** Comparison of ATFL thickness ratios and clinical outcomes between Grade 2 and Grade 3 ankle sprains.

Variable	Grade 2 (*n* = 17)	Grade 3 (*n* = 15)	*p*‐value
ATFL ratio (baseline)	1.48 ± 0.13	1.58 ± 0.20	0.220
ATFL ratio (3 weeks)	1.45 ± 0.16	1.39 ± 0.19	0.509
ATFL ratio (6 weeks)	1.30 ± 0.24	1.30 ± 0.17	0.650
ATFL ratio (12 weeks)	1.23 ± 0.16	1.20 ± 0.16	0.692
VAS (12 weeks)	1.06 ± 1.39	1.13 ± 1.41	0.871
AOFAS (12 weeks)	91.88 ± 8.02	90.27 ± 8.59	0.445
CAIT (12 weeks)	27.47 ± 1.94	27.00 ± 2.33	0.846

## 4 Discussion

The principal finding of this study was that temporal changes in anterior talofibular ligament (ATFL) thickness, quantified using an injured‐to‐healthy thickness ratio, were not significantly correlated with functional clinical outcomes at 12 weeks following acute lateral ankle sprain. Although ultrasonography demonstrated progressive morphologic changes in ATFL during the healing period, these structural changes did not reliably reflect patient‐reported recovery.

In the present study, the ATFL thickness ratio decreased significantly over time, suggesting ongoing structural remodeling after injury. This finding is consistent with the biological process of ligament healing, which involves an initial inflammatory phase followed by proliferation and collagen remodeling [18, 19]. However, despite these measurable structural changes, no significant correlations were observed between the ATFL thickness ratio and functional outcomes, including VAS, AOFAS, and CAIT scores. These results indicate that ligament thickness alone may not adequately represent functional recovery. In addition, the CAIT score was used as a patient‐reported measure of perceived ankle function and subjective instability during the early recovery period rather than as a diagnostic indicator of established chronic ankle instability. Because chronic ankle instability is typically defined over a longer follow‐up period, the 12‐week assessment in this study should be interpreted as reflecting short‐term functional recovery rather than long‐term mechanical instability.

Our findings are consistent with previous studies demonstrating that ligament thickening after ankle sprain reflects structural alteration rather than functional instability [4, 13, 15]. Prior cross‐sectional studies have shown that previously injured ankles exhibit increased ATFL thickness compared with uninjured ankles, yet similar degrees of thickening are observed in both asymptomatic copers and patients with chronic ankle instability [4, 6]. These observations suggest that morphologic changes alone cannot explain differences in symptoms or perceived instability. The present study extends these findings by demonstrating that even during the early healing phase after acute injury, serial changes in ligament thickness were not significantly associated with short‐term clinical outcomes.

One possible explanation for the lack of correlation between ligament thickness and functional outcome lies in the biological characteristics of ligament healing. Following injury, fibroblasts initially produce a relatively disorganized collagen matrix predominantly composed of Type III collagen, resulting in scar formation and increased tissue volume [4, 13, 15, 20, 21]. As healing progresses, collagen fibers gradually reorganize and align parallel to the direction of mechanical stress, leading to a more compact ligament structure and progressive reduction in thickness [20, 21, 22]. However, these morphological changes do not necessarily indicate restoration of normal biomechanical properties [4, 6]. Ligament healing is a complex biological process involving collagen reorganization, scar maturation, and recovery of tensile properties [4, 6, 21], which cannot be fully evaluated by thickness measurements alone [18, 21, 22]. Therefore, ultrasonographic morphology may not directly correspond to functional recovery or patient‐reported symptoms, and imaging findings should be interpreted together with clinical and functional assessment.

In addition, ankle stability is inherently multifactorial. Functional recovery after ankle sprain is influenced not only by static ligament integrity but also by neuromuscular control, proprioception, and peroneal muscle function [1, 3, 18, 20]. In this study, neither ATFL thickness ratio nor injury severity (Grade 2 vs. Grade 3) was associated with clinical outcomes at 12 weeks, suggesting that factors beyond structural ligament changes and initial injury severity may also contribute to functional recovery.

Collectively, these findings indicate a mismatch between structural healing and clinical recovery. Although ATFL thickness decreased over time, improvements in pain, function, and perceived stability did not parallel these changes. This highlights the limitation of relying on static imaging parameters as indicators of functional outcomes [19, 23].

This study has several limitations. First, the sample size was relatively small, which may have limited statistical power and increased the risk of Type II error. Therefore, weak associations between ATFL morphology and functional outcomes may not have been detected, and the absence of statistically significant findings should be interpreted with caution. Second, ultrasonographic evaluation focused primarily on ATFL thickness at the ligament midpoint, whereas insertional regions and other potentially relevant features such as fiber continuity, ligament tissue quality, and dynamic instability were not quantitatively assessed. Third, the follow‐up period was limited to 12 weeks and therefore only reflects early functional recovery after acute injury. Longer follow‐up would be necessary to evaluate long‐term outcomes and the potential development of chronic ankle instability. Finally, the exclusion of Grade 1 sprains and avulsion‐type injuries, as well as the limited number of older patients, may restrict the generalizability of the findings.

Although serial ultrasonography demonstrated progressive morphological remodeling of the ATFL following acute lateral ankle sprain, the present study did not detect a significant association between the ATFL thickness ratio and short‐term functional outcomes. Ultrasonographic thickness alone may not fully reflect functional recovery and should be interpreted together with clinical assessment.

## Author Contributions

**Seung Myung Wi:** conceptualization, methodology, writing – original draft. **Dong Hee Kim:** formal analysis, methodology, writing – original draft. **Sang Heun Kim:** data curation, resources. **Sung Jin Shin:** conceptualization, methodology, investigation, data curation, formal analysis, writing – original draft, writing – review and editing.

## Funding

The authors have nothing to report.

## Ethics Statement

This study was conducted in accordance with the Declaration of Helsinki. The study protocol was approved by the Institutional Review Board (IRB No. SCMC 2025‐12‐001).

## Consent

The requirement for informed consent was waived because of the retrospective nature of the study and the use of anonymized clinical data.

## Conflicts of Interest

The authors declare no conflicts of interest.

## Data Availability

The datasets generated and analyzed during the current study are not publicly available because they contain potentially identifiable patient information but are available from the corresponding author on reasonable request.
